# The Role of Clonal Hematopoiesis of Indeterminant Potential and DNA (Cytosine-5)-Methyltransferase Dysregulation in Pulmonary Arterial Hypertension and Other Cardiovascular Diseases

**DOI:** 10.3390/cells12212528

**Published:** 2023-10-26

**Authors:** Isaac M. Emon, Ruaa Al-Qazazi, Michael J. Rauh, Stephen L. Archer

**Affiliations:** 1 Department of Medicine, Queen’s University, Kingston, ON K7L 3N6, Canada; 17ime@queensu.ca (I.M.E.); 17rasa1@queensu.ca (R.A.-Q.); 2 Department of Pathology and Molecular Medicine, Queen’s University, Kingston, ON K7L 3N6, Canada; rauhm@queensu.ca

**Keywords:** DNA methylation, epigenetic regulation, inflammation, group 1 pulmonary hypertension, diabetes, congenital heart disease, Tatton-Brown-Rahman Syndrome

## Abstract

DNA methylation is an epigenetic mechanism that regulates gene expression without altering gene sequences in health and disease. DNA methyltransferases (DNMTs) are enzymes responsible for DNA methylation, and their dysregulation is both a pathogenic mechanism of disease and a therapeutic target. DNMTs change gene expression by methylating CpG islands within exonic and intergenic DNA regions, which typically reduces gene transcription. Initially, mutations in the *DNMT* genes and pathologic DNMT protein expression were found to cause hematologic diseases, like myeloproliferative disease and acute myeloid leukemia, but recently they have been shown to promote cardiovascular diseases, including coronary artery disease and pulmonary hypertension. We reviewed the regulation and functions of DNMTs, with an emphasis on somatic mutations in *DNMT3A*, a common cause of clonal hematopoiesis of indeterminant potential (CHIP) that may also be involved in the development of pulmonary arterial hypertension (PAH). Accumulation of somatic mutations in *DNMT3A* and other CHIP genes in hematopoietic cells and cardiovascular tissues creates an inflammatory environment that promotes cardiopulmonary diseases, even in the absence of hematologic disease. This review summarized the current understanding of the roles of DNMTs in maintenance and de novo methylation that contribute to the pathogenesis of cardiovascular diseases, including PAH.

## 1. Introduction

All cells in multicellular organisms contain the same genetic makeup, though the structure and function of these cells substantially differ between tissues. One mechanism that accounts for tissue heterogeneity in gene expression is ‘epigenetic regulation’. Epigenetic mechanisms are defined as those which regulate gene expression without altering DNA sequences. Epigenetic mechanisms mediate cell-specific plasticity in cellular phenotype and function, both in the context of health and disease, and are responsible for many aspects of the gene–environment interaction [1]. Epigenetic mechanisms likely underlie the ‘Barker hypothesis’, which proposed that adverse environmental exposures during fetal and neonatal development, such as intrauterine growth restriction and prematurity, can cause irreversible alterations to cell function that manifest as diseases later in life, such as coronary artery disease, type 2 diabetes, and hypertension [2]. Epigenetic modifications in response to environmental stimuli and toxins may initiate and maintain changes in gene expression that alter cell function and lead to disease [2]. 

There are three primary mechanisms of epigenetic regulation, including DNA methylation and demethylation, histone modification, and regulatory non-coding RNA activity [3]. Each of these processes are regulated, and there are important interactions amongst these three epigenetic mechanisms and between these epigenetic mechanisms and transcription factors in the regulation of genes [3]. Dysregulation of any of these mechanisms can have drastic implications on DNA transcription and cell function that can lead to a variety of diseases, including cancer, neurodegenerative disorders, and cardiovascular disease [4,5,6]. In this review, we focused on the role of DNA (cytosine-5) methyltransferases (DNMTs) in pulmonary arterial hypertension and cardiovascular disease (CVD).

## 2. DNA Methylation

DNA methylation, the transfer of a methyl group to a DNA nucleotide, is a major mechanism by which gene expression is regulated in health and disease. Fetal genes are often unmethylated during development but become methylated, turning off the fetal gene package. These fetal gene packages may be reactivated in diseases later in life. One of the most common mechanisms of DNA methylation is the addition of a methyl group to the 5th carbon on a cytosine nucleotide, generally one next to a guanine nucleotide (CpG) [7]. Though CpGs are relatively scarce in the human genome, with less than 1% of all adjacent nucleotides being CpGs, approximately 60–80% of all CpGs are methylated [7]. Over 90% of CpGs are scattered throughout the genome, while less than 10% occur in a clustered manner. Clusters, which have a dense content of CpG dinucleotides, are referred to as CpG islands [7,8]. CpG islands are commonly found within gene promoter and enhancer regions and are defined as having greater than 200 base-pair regions with a greater than 50% CG content [9]. CpG islands are predominantly hypomethylated, and are generally located upstream of housekeeping genes that are important for maintenance and function across cell types [8]. Default hypomethylation permits ready transcription of these genes. When CpG islands are highly methylated, the chromatin in the region of the gene becomes compacted and transcription is reduced as a result [10]. A greater GC content provides more opportunities for DNA methylation to regulate gene transcription and potentially disrupt normal cell development and function. 

Since 1992, gene methylation status has been measurable using bisulphite genomic sequencing [11]. In bisulfite polymerase chain reaction (PCR), DNA is first denatured and then exposed to bisulfite before PCR and sequencing. Methylated cytosine nucleotides are resistant to bisulfite degradation and are sequenced as cytosine; in contrast, unmethylated cytosines are converted into uracil residues, which, in the subsequent PCR amplification and sequencing steps, become thymine residues [11]. It is now possible to map the entirety of the methylome at each stage of the cell’s life, allowing us to understand how the dysregulation of methylation can lead to the manifestation of diseases [12]. There are two major high-throughput techniques to evaluate the methylome, meaning the methylation status of all cytosines in the genome; these techniques are whole-genome bisulfite sequencing (WGBS) and reduced representation bisulfite sequencing (RRBS) [12,13]. In RRBS, GC-enriched DNA is preferentially isolated via enzymatic digestion with reagents that generate fragments with CpG dinucleotides at both ends, regardless of whether these segments are methylated. This assay then isolates short DNA fragments, largely GC-rich regions, discarding the larger fragments, which are not GC-rich and are thus less likely to be methylated as a result [14]. RRBS identifies most of the methylome, with lower requirements for total DNA than conventional bisulfite sequencing; however, not all of the methylome is revealed, as this assay excludes large DNA segments [14]. In contrast, WGBS is similar to conventional bisulfite PCR but involves the creation of a DNA library that is treated with bisulfite and then analyzed via next-generation sequencing (NGS), rather than conventional PCR, prior to high-throughput analysis [14].

## 3. DNA Methyltransferases

There are five DNMTs encoded by the human genome: DNMT1, DNMT2, DNMT3-Alpha (DNMT3A), DNMT3-Beta (DNMT3B), and DNMT3-like (DNMT3L). DNMT1, DNMT3A, and DNMT3B are the three main enzymes that are responsible for CpG methylation [7,8]. They are vital in ensuring normal cell development and mediating responses to environmental factors, such as hypoxia, poor nutrition, and toxin exposure. DNMT1 maintains the methylome in the long term and ensures consistency in the regulation of developmental genes throughout life [15]. When cells undergo mitosis, the DNA methylation pattern is maintained in daughter cells mainly via DNMT1, which mediates heritable, epigenetic regulation. However, the consistency of the heritability of the methylome may be lost with increased repetition of cell division [16]. 

DNMT3A and DNMT3B are responsible for de novo methylation and the activation or silencing of genes important for cell adaptation and differentiation [15]. DNMT2 is responsible for post-transcriptional regulation via RNA methylation [17], while DNMT3L is an inactive DNMT that acts as a cofactor for DNMT3A and DNMT3B activity [17]. DNMT2 and DNMT3L are suspected to play less of a role than the other DNMTs in disease-related epigenetic regulation.

**Figure 1:**
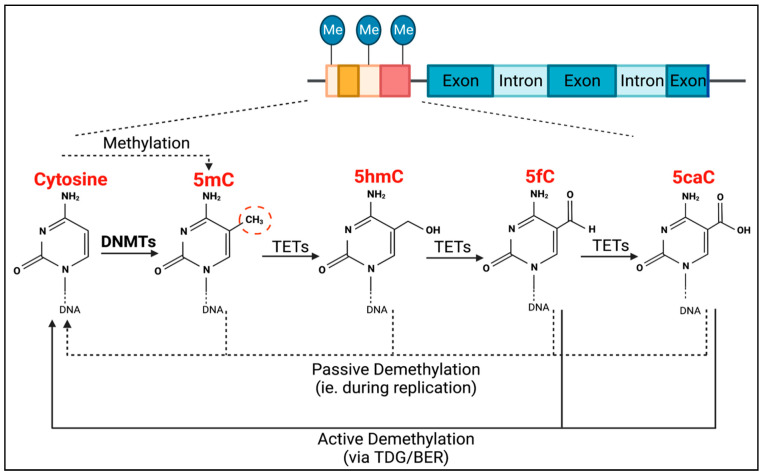
Process of DNMT- and TET-mediated regulation of DNA methylation. DNMTs are responsible for CpG methylation (dashed red circle) of guanosine-adjacent cytosines, forming 5-methylcytosine (5mC). TETs are then responsible for the process of demethylation. They mediate the conversion of 5mC into 5-hydroxymethylcytosine (5hmC), 5hmC into 5-formylcytosine (5fC), and 5fC into 5-carboxylcytosine (5caC). Both 5fC and 5caC can be actively demethylated to cytosine via thymine DNA glycosylase (TDG)-mediated base excision repair (BER) (solid black lines). Passive demethylation, due to DNA replication, can also occur at any stage, resulting in non-methylated cytosine (dotted lines).

TET methylcytosine dioxygenase 2 (TET2) is the enzyme responsible for the active removal of methyl groups, which is completed via thymine DNA glycosylase (TDG)-mediated base excision repair (BER) (Figure 1) [18]. TET2 facilitates the hydroxylation of 5-methylcytosine to become 5-hydroxymethylcytosine, as well as the subsequent oxidation steps to produce 5-formylcytosine, and then 5-carboxylcytosine (Figure 1). Passive demethylation can also occur at any stage during DNA replication (Figure 1). 

Changes in the epigenetic or genetic regulation via DNMTs or TET2 can contribute to downstream gene dysregulation and alterations in cellular protein function, leading to disease. This review was inspired by published research in our laboratory that identified somatic and germline mutations of *TET2* as predisposing to inflammation [19] and PAH [20] and emerging research, suggesting that somatic mutations in *DNMT3A* may also promote inflammation and PAH. Mutations of *TET2* and *DNMT3A* are both established drivers of clonal hematopoiesis of indeterminate potential (CHIP) [21]. We are intrigued by the observation that the loss of TET2 function, which would be predicted to increase gene methylation, results in similar endpoints (development of inflammation and PAH) as those that occur with loss-of-function mutations of *DNMT3A*, which might be expected to reduce gene methylation.

## 4. Clonal Hematopoiesis of Indeterminate Potential

**Figure 2:**
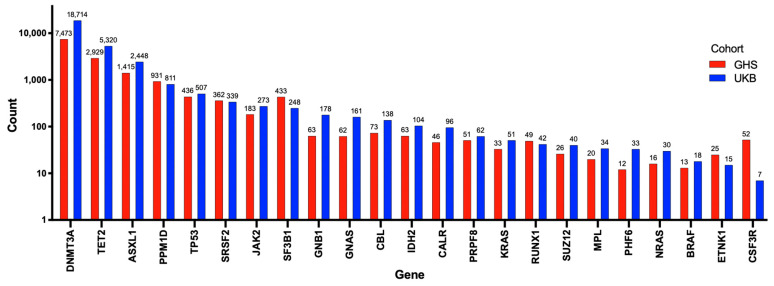
Number of individuals with mutations in each of the 23 genes used to identify CHIP. Adapted from Kessler et al. (2021) [24]. In total, 40,208 patients were identified with CHIP using exome sequencing data on 628,388 individuals from the UK biobank and Geisinger Health System. The number of individuals with each mutation is exponentially presented on the Y-axis, and the 23 genes used to determine CHIP are shown on the X-axis. Individuals from the Geisinger Health System are represented by red bars, and those from the UK biobank are represented by blue bars. *DNMT3A*, *TET2*, and *ASXL1* were the 3 most commonly mutated genes identified.

To better understand the potential role of mutations of *DNMT3A* in cardiovascular diseases, including PAH, it is useful to review its more established role as a driver of CHIP. Somatic mutations in hematopoietic stem cells (HSCs), such as *DNMT3A* mutations, lead to hematologic malignancies, especially in the myeloid lineage [22]. CHIP is defined as the earliest, pre-malignant state, when an HSC acquires an advantageous somatic mutation (most often in just *DNMT3A* or *TET2*) that becomes detectable in expanded mutant progeny blood cells at an allele frequency of at least 2% [22]. The burden of mutations causing CHIP increases with age, reflecting the cumulative deleterious effects of aging and mutagens on the genome. CHIP is present in over 10% of individuals over the age of 65 years, rapidly increasing in prevalence to 30% in people over the age of 85 years [22]. However, clones with lower variant allele frequencies (VAF < 2%) can be found in 95% of individuals over the age of 50 years [22]. Mutations in several genes have been associated with the development of CHIP, though *DNMT3A*, *TET2*, and *additional sex combs like-1* (*ASXL1*) have been consistently reported as the most frequently mutated genes (Figure 2) [23,24]. Kessler et al. used exome sequencing data from 628,388 individuals from the UK Biobank (UKB) and Geisinger Health System (GHS) and identified 40,208 (6.37%) carriers of CHIP (Figure 2) [24]. The number of patients possessing a mutation in each CHIP driver gene is shown in Figure 2 [24]. *DNMT3A* was the most frequently mutated gene, with approximately 70% of patients with CHIP having a *DNMT3A* mutation (Figure 2) [24]. 

CHIP mutations provide the affected cell with a reproductive advantage and allow for a disproportionate rate of self-renewal, resulting in clonal proliferation [25]. The presence of these clones can affect the functionality of the immune system, most notably favouring inflammation and the development of diseases, like myelodysplastic syndromes (MDSs) and acute myeloid leukemia (AML) [22,25]. CHIP is not yet screened for routinely, though it may be incidentally discovered in solid tumour patients who have their blood sequenced as a control or in some direct-to-consumer sequencing instances. Additionally, CHIP may be uncovered in patients with unexplained cytopenia not meeting the criteria for MDSs, specifically referred to as clonal cytopenia of undetermined significance [26]. There are two main approaches to identifying CHIP. Firstly, CHIP may be detected via targeted sequencing using commercial panels, such as the Thermo Fisher Oncomine Myeloid Next-Generation Sequencing Panel, which targets 40 genes and 29 fusion drivers (RNA that initiates the production of novel fusion proteins [27]) that are involved in myeloid malignancies [28]. Alternatively, CHIP can be found by mining existing whole genome sequencing or whole exome sequencing data, as described by the authors of [29]. Though cells at the CHIP stage often only possess one driver mutation, it is the accumulation of mutations in an expanded clone that leads to diseases, such as MDSs vs. AML. In addition, disease stage/severity may reflect which CHIP genes are mutated [30]. 

**Figure 3:**
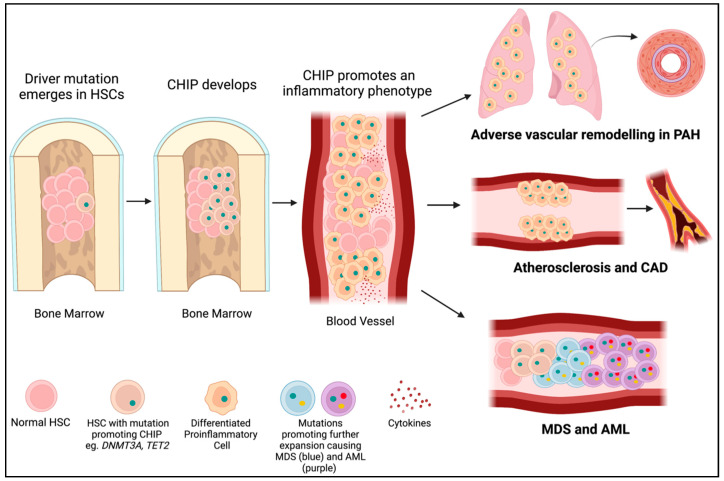
CHIP mutations lead to PAH, cardiovascular disease, and cancer. A driver mutation (green dot) occurs in an HSC in the bone marrow (often in *DNMT3A* or *TET2*). If it provides a selective advantage for the cell, it will proliferate into a clone (CHIP). CHIP promotes the differentiation of HSCs into immune cells with an inflammatory phenotype. These inflammatory cells may infiltrate the lungs and contribute to PAH or embed in the endothelium and contribute to atherosclerosis and CAD. Further mutations (yellow and red dots) in hematopoietic precursor cells in the bone marrow may lead to the development of myelodysplastic syndromes (blue cells) and acute myeloid leukemia (purple cells).

*DNMT3A* is the most commonly mutated gene in CHIP [31], accounting for approximately 25% of mutations in patients with CHIP who develop leukemia [25,32]. Somatic *DNMT3A* mutations in HSCs also increase the risk of developing CVD by promoting a pro-inflammatory phenotype, marked by the elevation of numerous cytokines, such as interleukin 1ß (IL1ß) (Figure 3). Mutations in *DNMT3A* and *TET2* account for approximately 50% of CHIP mutations in people with atherosclerosis, with somatic *DNMT3A* mutations accounting for roughly 35% of mutations in patients with CHIP and coronary artery disease and early-onset myocardial infarction [33,34]. The bulk of this review discussed the roles of DNMT1, DNMT3A, and DNMT3B in both germline and hematopoietic cells in the development of PAH and cardiovascular diseases.

## 5. DNMTs in Pulmonary Arterial Hypertension

PAH is a fatal cardiopulmonary disease characterized by adverse vascular remodelling of small pulmonary arteries, restricting blood flow through the pulmonary circulation, as reviewed by the authors of [35]. The increased pulmonary vascular resistance increases afterload on the right ventricle (RV), initially leading to compensatory right ventricular hypertrophy (RVH), but ultimately to RV fibrosis, dilatation, and heart failure. Patients develop dyspnea and impaired exercise tolerance, and many die of RV failure. The genetic basis of PAH has been well established, with ~20 known genes that cause PAH. Approximately 10–20% of patients with idiopathic PAH have a pathologic gene mutation versus a 70% mutation prevalence in patients with familial PAH. However, these gene mutations have yet to be described, with a significant frequency in the ~45% of PAH patients with associated PAH (meaning PAH associated with connective tissue diseases). The first described PAH gene was *BMPR2*, and this mutation continues to account for the majority of all mutations in PAH patients. Subsequently, many genes have been identified that predispose to PAH, most of which are inherited as autosomal dominant mutations with variable penetrance, including BMP/TGF-β family members (*ACVRL1*, *BMPR1B*, *GDF2*, *ENG*, *CAV1*, *SMAD1*, *SMAD4*, and *SMAD9*), ion channels and transporters (*KCNK3*, *KCNA5*, *ATP13A3,* and *AQP1*), transcription factors (*SOX17*, *TBX4*, and *KLF4*), miscellaneous genes (*KDR*, *PTGIS*, *RNF213*, *FBLN2*, and *PDGFD*), as well as the demethylation regulator, *TET2*. *EIF2AK4*, which predisposes to pulmonary veno-occlusive disease, is unique in having autosomal recessive inheritance.

**Figure 4:**
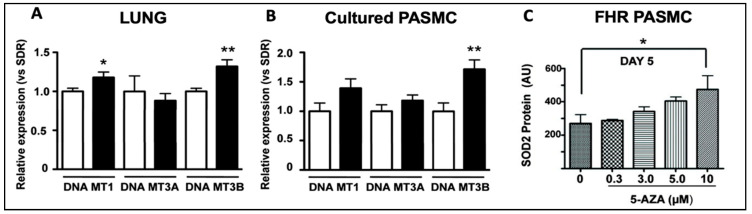
DNMT1 and DNMT3B mRNA are increased in the lungs of rats with PAH and in PASMCs. Reproduced from the study published by the authors of [38]. (**A**) DNMT1 and DNMT3B mRNA were increased in the lungs of FHRs (black bars), which spontaneously develop PAH, compared to control Sprague Dawley rats (SDR) (*n* = 12 in each group). (**B**). In PASMCs, FHRs displayed higher levels of DNMT3B expression, along with a trend towards higher DNMT1 and DNMT3A expression than SDRs. (**C**) SOD2 expression was restored in FHR PASMCs that received 10 μm of 5-AZA, a DNMT inhibitor, reducing proliferation and enhancing apoptosis. * *p* < 0.05 and ** *p* = 0.01.

While the role of *TET2* mutations has been reported [20] and confirmed [36,37] in PAH patients, the role of *DNMT3A* mutations remains unknown. A role for DNMTs in the epigenetic basis of PAH was described in 2010 [38]. This study noted that DNMT 1 and 3B upregulation led to pathologic methylation and a partial silencing of mitochondrial superoxide dismutase (SOD2) [38]. The resulting alteration of SOD2 function and mitochondrial redox signalling caused the pathologic activation of HIF-1α during normoxia, creating a cancer-like hyperproliferative PASMC phenotype [38]. Bisulfite sequencing demonstrated the selective hypermethylation of a CpG island in an enhancer region of intron 2 and another in the promoter of SOD2 [38]. The resulting decrease in SOD2 expression was only found in pulmonary artery smooth muscle cells (PASMCs), not in aortic smooth muscle cells [38], reflecting tissue heterogeneity in the epigenetic regulation of gene expression. Moreover, the reduction in pulmonary artery SOD2 expression, also seen in patients with PAH, was associated with changes in redox signalling, notably diminishing H_2_O_2_ production [38]. The altered redox milieu activated HIF-1α, creating a state which mimics hypoxia despite the abundant oxygen availability, and promoting a state of pseudohypoxia in animals and humans [38]. This study found that levels of both DNMT1 and DNMT3B were upregulated in the lungs of the fawn-hooded rat, a rat model in which PAH develops spontaneously (Figure 4) [38]. The DNMT inhibitor 5-aza-2′-deoxycytidine (Decitabine^®^), which is used in patients to treat myelodysplastic syndrome [39], restored SOD2 expression, suggesting that dysregulated DNMT activity may promote the excessive proliferation and impaired apoptosis of PASMCs in PAH [38]. The administration of 5-aza-2′-deoxycytidine (in PASMCs), or the administration of SOD analogs (in vivo), corrected the proliferation-to-apoptosis ratio, which was elevated (favouring proliferation) in PAH [38].

**Figure 5:**
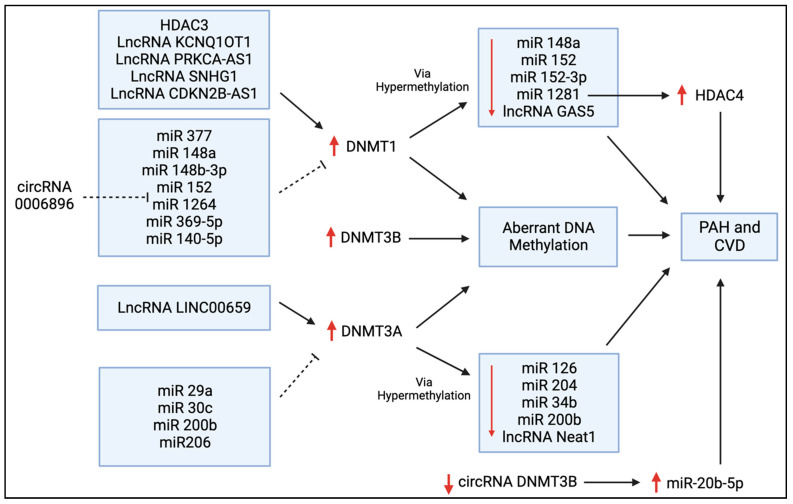
Regulators of DNMT expression in PAH and CVD. This schematic diagram shows the bi-directional regulation of DNMT expression via histone deacetylases (HDACs), long non-coding RNAs (lncRNAs), microRNAs (miRs), and circular RNAs (circRNAs) that have all been discussed in this review. The upregulation of these DNMTs leads to aberrant DNA methylation, and this can ultimately contribute to the pathogenesis of PAH and other forms of CVD. DNMTs are often upregulated by lncRNAs and downregulated by a variety of miRs, as shown here. Conversely, the suppressive effects of DNMT1 and DNMT3A on several miRs are also shown.

Zhang et al. reported that miR-140-5p is downregulated in hyperproliferative diseases, such as PAH, leading to adverse vascular remodelling [40]. They reported that a synthetic miR-140-5p targets DNMT1 mRNA and prevents DNMT1-mediated hypermethylation (Figure 5), thereby increasing the expression of SOD2, which inhibits the hypoxia-mediated proliferation of human PASMCs and promotes apoptosis and differentiation [40]. Potus et al. found that DNMT3A and DNMT3B were upregulated in the RVs of PAH patients with decompensated RV failure [41]. This resulted in the hypermethylation of the miR-126 promoter (Figure 5) and, subsequently, reduced miR-126 expression, leading to decreased angiogenesis and RV vascular density, contributing to RV failure [41]. They also found that the treatment of decompensated RV endothelial cells with hydralazine, a DNMT inhibitor, increased miR-126, and that miR-126 upregulation improved RV microcirculation and RV function in a MCT-induced rat model of PAH [41]. In a further example of the interrelatedness of epigenetic mechanisms, Li et al. found that *DNMT1* upregulation reduces miR-1281 expression, which, in turn, enhances histone deacetylase 4 (HDAC4) activity (Figure 5), ultimately increasing PASMC proliferation and adverse vascular remodelling [42]. Thus, it may be feasible to directly target DNMTs as a therapy for PAH or indirectly target DNMTs by manipulating miRs or HDACs. 

Tian et al. linked the Warburg shift in mitochondrial metabolism and the hyperproliferative phenotype of right ventricular fibroblasts in MCT PAH to upregulated DNMT1 expression [43]. DNMT1 expression was elevated due, in part, to a decrease in its regulatory microRNA, miR-148b-3p [43]. DNMT1 upregulation caused pathologic, normoxic HIF-1α activation, which increased the expression of pyruvate dehydrogenase (PDH) kinases (PDK1 and PDK3), thereby inhibiting PDH and impairing oxidative metabolism [43]. siRNAs targeting PDK1 and PDK3 restored mitochondrial superoxide and hydrogen peroxide production and inactivated HIF-1α [43]. This indicates that the epigenetic regulation of DNMTs can regulate metabolism and can contribute to the pseudohypoxic state in RV fibroblasts in experimental PAH. This study concluded that in experimental PAH, RV fibroblasts manifest a DNMT1-HIF-1α-PDK-mediated, chamber-specific, epigenetically regulated metabolic memory that promotes their rapid proliferation and results in increased collagen production and RV fibrosis.

While the upregulation of DNMTs is most often pathologic, there are limited reports of a beneficial effect of DNMT upregulation. For example, Yan et al. showed that in rodent PAH induced via either MCT or hypoxia, DNMT3B protein and mRNA were increased in the lungs, a finding also noted in the PASMCs of PAH patients, which was assumed to be compensatory [44]. Furthermore, *DNMT3B^−^*^/*−*^ rats displayed severe adverse pulmonary vascular remodelling [44]. Inhibition of DNMT3B promoted PASMC proliferation, while overexpression attenuated migration [44]. Overexpression of DNMT3B was also found to transcriptionally regulate inflammatory pathways, and could attenuate hypoxia-induced PH and right ventricular hypertrophy in mice, demonstrating the therapeutic potential of upregulating DNMT3B in the treatment of PH [44]. 

**Figure 6:**
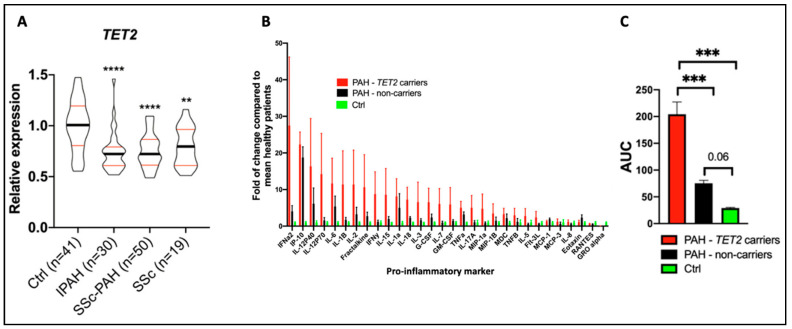
Reduced TET2 expression and *TET2* mutations are associated with PAH and inflammation in patients. These data were published by our group in Circulation in 2020 [20]. (**A**) *TET2* expression in peripheral blood mononuclear cells was compared between 41 healthy controls, 30 IPAH patients, 50 scleroderma-associated PAH patients, and 19 patients with scleroderma and no PAH. *TET2* expression was reduced in PAH patients compared to controls. Expression levels were also reduced in patients with scleroderma. ** *p* < 0.01 and **** *p* < 0.0001. (**B**) The expression of 30 cytokines was measured in PAH patients with (*n* = 10) and without (*n* = 10) *TET2* mutations and in 9 healthy control patients. An increased expression of 28 cytokines was found in individuals with *TET2* mutations. The values presented are fold change compared to healthy patients. (**C**) Patients with *TET2* mutations displayed increased levels of pro-inflammatory markers, as measured via an increase in the area under the curve (AUC). *** *p* < 0.001.

While the expression of DNMTs is often regulated via transcription factors or epigenetic mediators, mutations (somatic or germline) can also change the function and expression of these methyltransferases. In fact, Huang et al. found that 74% of 253 disease-associated *DNMT3A* mutations were loss-of-function mutations, with half of these variants displaying reduced protein stability [45]. We have previously shown that *TET2* is a human PAH gene [20], and are continuing to evaluate *DNMT3A* as a human PAH gene, a finding we reported in abstract form in the year 2019 [46]. We found that both *DNMT3A* and *TET2* germline deleterious mutations were significantly associated with PAH in European individuals, and that several additional cases contained splice variants and CHIP mutations in these genes [46]. We have also found reduced TET2 expression levels in PAH patients compared to controls and demonstrated that mutations in *TET2* promote an inflammatory form of PAH in patients, characterized by an increase in circulating levels of inflammatory cytokines, including IL-6 and IL-1β (Figure 6A–C) [20]. Using the PAH Biobank, we showed that 0.39% of the study patients (10/2572) had 12 *TET2* mutations (75% predicted germline and 25% somatic), indicating that *TET2* is a probable PAH gene [20]. *TET2* mutations were associated with idiopathic PAH (7/812 patients, relative risk: 10.79, *p* = 8.483 × 10^−5^) [20].

Supporting the human genetic findings and adding biologic plausibility was the observation, in the same publication, that a murine model, in which *Tet2* is deleted in hematopoietic cells, spontaneously developed PAH and inflammation [20]. As in patients with *TET2* mutations, the PAH in these *Tet2^−^*^/*−*^ mice was associated with a substantial increase in circulating cytokine levels, including IL-1β. Consistent with the pathologic contribution of this inflammatory state to the development of PAH, mice with a homozygous deletion of TET2 responded therapeutically with reduced PAH to therapy with canakinumab, an IL-1β inhibitory antibody [20]. This observation also suggests a potential role for IL-1β-targeted therapies in PAH. This finding in PAH is analogous to earlier observations of the importance of this CHIP gene in patients with atherosclerosis and a persistent pro-inflammatory response. In the CANTOS study, which included 10,061 patients with prior myocardial infarction and elevated levels of C-reactive protein, indicating the patients were inflamed, canakinumab was also found to be therapeutic [47]. In the CANTOS study, canakinumab was administered at several doses every 3 months for 48 months. Canakinumab (150 mg) subcutaneously met the prespecified primary efficacy endpoint, reducing non-fatal myocardial infarction, non-fatal stroke, and cardiovascular death, independent of the effects of lipids, highlighting the pathologic role of inflammation in ischemic heart disease and stroke. In the CANTOS cohort, 338 patients (8.6%) had clonal hematopoiesis variants, with 103 patients and 85 patients having variants in *TET2* and *DNMT3A*, respectively [48]. Patients with CHIP and a somatic *TET2* mutation appeared to benefit more from canakinumab than did study participants without CHIP [48]. It is unknown whether patients with atherosclerosis (or PAH) should be screened for *TET2* mutations, particularly as canakinumab is not currently approved for either indication. This drug has been approved for treating Still’s disease, an autoimmune condition characterized by arthritis, fever, and rash [49]. The use of this biologic therapy has been associated with an increased risk of infection.

The *TET2*-PAH discovery is an interesting example of a genetically encoded disorder of epigenetics mediated via the loss of function of a gene that regulates gene demethylation [20]. This work established *TET2* as a new PAH gene, which was subsequently confirmed by a group in Japan [36]. We did not establish whether the germline *TET2* mutations were inherited or were acquired de novo, as is known to occur with a number of PAH genes, particularly in the paediatric PAH population [50].

A recent study found upregulated *DNMT1* and *DNMT3B* expression and increased DNMT activity in the lungs of rats with sugen5416/hypoxia-induced pulmonary hypertension [51]. Jacob et al. also revealed a link between metabolism and DNMT regulation. They found that glucose-6-phosphate dehydrogenase (G6PD), the rate-limiting enzyme in the pentose phosphate pathway (which is upregulated under pulmonary hypertension), upregulates DNMT activity, which decreases the expression of genes encoding vascular-protective proteins, including SOD2 and nitric oxide synthase 3 (NOS3), ultimately promoting the pathogenesis of PH [51]. Thus, several studies have demonstrated the association between upregulated DNMT expression and PAH; however, it is unclear whether and how mutations in DNMT genes, including CHIP-driving *DNMT3A* mutations, cause PAH, and this remains an active area of investigation in our laboratory. We hypothesized that, as with *TET2* mutations, *DNMT3A* mutations may cause PAH by elevating inflammation. These studies of *DNMT3A* are particularly relevant to the cohort of patients with associated PAH (APAH), a group that is primarily comprised of female patients with scleroderma, CREST syndrome, or systemic lupus, as these patients are inflamed, and no gene has yet been identified as being enriched in this cohort, which accounts for ~40% of all group 1 PH. However, CHIP has been significantly associated with systemic sclerosis in patients under 50 years of age, with *DNMT3A* being the most frequently mutated gene [52]. Further, David et al. identified an association between CHIP and systemic lupus erythematosus (SLE), noting that CHIP developed over 20 years earlier in SLE patients compared to controls (*p* < 0.00001) [53]. Once again, most mutations occurred in the *DNMT3A* gene [53], suggesting a potential connection between *DNMT3A*-driven CHIP and both SLE and systemic sclerosis. 

## 6. DNMTs in Atherosclerosis, Coronary Artery Disease, and Myocardial Infarction

Atherosclerosis is a vascular disease characterized by intimal thickening and the formation of lipid-laden fatty streaks that progresses to the formation of atherosclerotic plaques, which are focal accumulations of lipids with high cholesterol content covered by a fibrous cap that intrudes into the arterial lumen [54]. These plaques often have a necrotic core with associated inflammation and may rupture, causing localized thrombosis that, in the heart, leads to acute coronary vascular occlusion and myocardial infarction. The roles of DNMTs in atherosclerosis have been extensively investigated, as they regulate key disease determinants, such as calcification, triglyceride accumulation, and inflammation [55,56,57,58,59,60,61]. Numerous studies have shown that DNMT-mediated hypermethylation of CpG islands is present in the promoter regions of genes that prevent calcium accumulation in the vascular wall [57,58,59]. Both miR-204 and miR-34b gene promoter regions are hypermethylated by DNMT3A in calcified vascular smooth muscle cells (VSMCs) (Figure 5) [58,59]. Knocking down DNMT3A restored the expression of both miR-204 and miR-34b and reduced the high phosphate-induced calcification of VSMCs [58,59]. This illustrates that changes in methylation can lead to downstream regulatory consequences that are only indirectly mediated via CpG methylation and are more directly related to the regulation of miRs via the altered transcription of intronic DNA. Furthermore, in patients with chronic kidney disease, indoxyl sulfate, a gut-derived uremic toxin, increases DNMT1- and DNMT3A-mediated hypermethylation of the promoter of the aging suppressor gene, Klotho, thereby promoting vascular calcification [57]. 

In addition to calcium buildup, excess cholesterol and inadequate breakdown of lipids can lead to atherosclerosis. DNMT1 plays a role in the upstream control of triglyceride metabolism [60]. In fact, miR-377 increases lipoprotein lipase activity, directly targeting the 3′-untranslated region (3′-UTR) of DNMT1 mRNA, inhibiting DNMT1 expression (Figure 5) [60]. This prevents hypermethylation of the glycosylphosphatidylinositol-anchored high-density lipoprotein-binding protein 1 (GPIHBP1) promoter, allowing for GPIHBP1 expression and binding to lipoprotein lipase, ultimately increasing triglyceride breakdown and preventing plaque buildup in an Apolipoprotein E (ApoE) murine knockout model of atherosclerosis [60]. Conversely, DNMT3B promotes lipid accumulation by interfering with the ability of the transcription factor specificity protein 1 (SP1) to bind to the scavenger receptor class B member 1 (SCARB1) promoter, thereby downregulating SCARB1, which is normally anti-atherosclerotic [61].

DNMT methylation is a major driver of the inflammatory nature of atherosclerotic plaques. Overexpression of DNMT1 in macrophages significantly increases pro-inflammatory cytokine production and accelerates atherosclerosis in ApoE KO mice [62]. DNMT1 methylates the promoter region of peroxisome proliferator-activated receptor gamma (PPAR-γ), downregulating its expression [62]. PPAR-γ is a member of the nuclear receptor superfamily and commonly plays an anti-inflammatory role by promoting polarization towards M2 macrophages, which are anti-inflammatory and promote tissue repair [62]. Moreover, DNMT1-induced inflammatory cytokine production is prevented by overexpressing PPAR-γ [62]. Conversely, patients with atherosclerosis have increased expression of DNMT1 and pro-inflammatory cytokines with decreased PPAR-γ expression [62]. DNMT1 also hypermethylates the promoter of atheroprotective Krüppel-like factor 4 (KLF4), downregulating its expression and promoting M1 macrophage inflammation and atherosclerosis [63]. 

Foam cells are fat-laden cells that have a M2 macrophage-like phenotype. They ingest lipids and reside within atherosclerotic vascular plaques. The disintegrin, ADAM metallopeptidase domain 10 (ADAM10), is upregulated in foam cells [64]. The long non-coding RNA (lncRNA), cyclin-dependent kinase inhibitor 2B antisense RNA 1 (CDKN2B-AS1), binds to and recruits DNMT1 to the ADAM10 promoter, hypermethylating the promoter and downregulating ADAM10, ultimately reducing the inflammatory response and promoting cholesterol efflux in atherosclerosis [64]. In addition to the lncRNA CDKN2B-AS1, DNMT1’s expression in foam cells is reciprocally regulated by miR-148a/152 (Figure 5), likely playing an essential role in homocysteine-related atherosclerosis [65]. Homocysteine induces atherosclerosis when present in excess, and hyperhomocysteinemia, induced by a diet high in the homocysteine precursor methionine, is often used to induce murine atherosclerosis models [66]. Homocysteine upregulates c-Myc [66], a transcription factor that upregulates the expression of DNMT1. One consequence of this homocysteine-dependent, c-Myc-mediated DNMT1 upregulation is the hypermethylation of the promotor of mitofusin-2 (MFN2), an anti-proliferative, large GTPase that mediates mitochondrial fusion [66]. MFN2 is an inhibitor of smooth muscle cell proliferation [67], and DNMT1-mediated inhibition of MFN2’s expression in VSMCs favours the proliferation and formation of atherosclerotic plaques [66]. Further, DNMT1 can translocate from the nucleus to the mitochondria, where it is responsible for the methylation of mitochondrial DNA (mtDNA), specifically methylating the mitochondrial displacement loop (D-loop methylation), which is responsible for mtDNA replication. In VSMCs, D-loop methylation via DNMT1 impairs mitochondrial function and decreases VSMC contraction in atherosclerosis [68]. 

Mutations in the *DNMT3A* and *TET2* genes also impair mtDNA integrity by regulating transcription factor A mitochondria (TFAM), a key player in mtDNA maintenance [69]. The loss of function of either gene results in decreased TFAM expression, leading to the intracellular release of mtDNA [69]. In macrophages, this activates cyclic GMP-AMP synthase (cGAS) and induces a type I interferon response, potentially promoting atherosclerosis [69]. CHIP, associated with *DNMT3A* and *TET2* mutations, also promotes activation of the nucleotide-binding domain, leucine-rich-containing family, pyrin domain-containing-3 (NLRP3) inflammasome in macrophages and increases the release of inflammatory cytokines, like IL-1β [70]. Increased inflammasome activity associated with TET2 CHIP in mice has been linked to an accelerated development of atherosclerosis [71] and greater cardiac dysfunction [72]. Macrophages are not the only cells in which epigenetic regulation promotes atherosclerosis. DNMT3B also accelerates atherosclerosis by hypermethylating the forkhead box P3 (FOXP3) promoter and decreasing FOXP3 expression in regulatory T cells (Tregs), which are cells that normally prevent inflammation and autoimmunity [73]. FOXP3 is an immunoregulatory DNA-binding protein that is vital in the development and function of Tregs, and the DNMT3B-mediated downregulation of FOXP3 induces atherosclerosis [73]. DNMTs may also promote atherosclerosis through other pathways. There is evidence that the circRNA-0006896-miR1264-DNMT1 axis (Figure 5) plays a role in destabilizing carotid plaque formation in atherosclerosis by promoting vascular endothelial cell proliferation, leading to plaque rupture [74]. Inhibiting DNMT1-dependent methylation of the estrogen receptor α (ERα) gene increases ERα expression and prevents post-menopausal atherosclerosis and homocysteine-induced endothelial cell apoptosis in mice [75]. Further, non-laminar blood flow can increase DNMT1-dependent DNA hypermethylation, reducing the expression of mechanosensitive genes, including *HoxA5* and *Klf3* in endothelial cells, favouring the development of atherosclerosis [56]. The DNMT inhibitor 5-AZA significantly reduced atherosclerosis in an ApoE^−/−^ mouse model [56]. 

Individuals with a variant *DNMT3A* gene (the rs13420827GG genotype) are at an increased risk of *Helicobacter pylori* infection, which may predispose to premature coronary artery disease (CAD) (odds ratio: 2.7) [76]. There is limited evidence that *Helicobacter pylori* infection is associated with the development of CAD [76,77,78]. This association was found using 561 patients with premature CAD compared to 599 healthy controls. The increased risk of *Helicobacter pylori* infection in patients with the *DNMT3A* rs13420827GG genotype demonstrates a potentially indirect way in which DNMT variants can promote CVD [76]. 

While we have reviewed the inflammatory and atherogenic consequences of pathologic DNMT activation, loss of DNMT expression and function can also have a detrimental impact. For example, extracellular matrix stiffening upregulates discoidin domain receptor 1 (DDR1), a receptor tyrosine kinase, resulting in DNMT1 downregulation via the ERK-p53 pathway [79]. Reduced DNMT1 activity results in the hypomethylation of regulatory regions of pro-inflammatory genes, enhancing the expression of VSMC inflammatory cytokines, such as MCP-1 and IL-6 [79]. Interestingly, genome-wide hypomethylation has been reported in CAD [80], and has also been seen in homocysteine-treated VSMCs, despite increased levels of DNMT3A and DNMT3B [81]. In contrast, we recently reported genome-wide hypermethylation of all chromosomes in patients with PAH, particularly those patients with *TET2* mutations [82]. High cholesterol and methionine levels can also reduce DNMT1 expression, leading to DNA hypomethylation [55]. 

While CHIP-associated *DNMT3A* and *TET2* mutations likely reduce enzymatic function, the consequences on biological processes of less versus more methylation are not necessarily opposed; instead, they depend on which targets are hypo- or hyper-methylated. For example, loss-of-function mutations of *DNMT3A* and *TET2* both promote the proliferation of HSCs through opposing mechanisms [83]. *DNMT3A* mutations are associated with loss of DNA methylation in regulatory regions near genes associated with HSC activity, such as homobox B3 (*HOXB3*), whereas *TET2* mutations are associated with gain of DNA methylation in regulatory regions associated with stem cell differentiation, such as the dedicators of cytokinesis 9 and 10 (*DOCK9* and *DOCK10*) [83]. Therefore, both *DNMT3A* and *TET2* mutations are associated with HSC proliferation and stem cell renewal, driving CHIP and its pro-inflammatory phenotype. 

CHIP mutations do not only occur in hematopoietic cells. Büttner et al. recently found that 88% of CHIP mutations (primarily in *DNMT3A* and *TET2*) present in the blood of patients with peripheral artery disease (PAD) were also present in atherosclerotic lesions, and that several patients also had these mutations in arterial collaterals, perivascular fat, and subcutaneous tissue [84]. This initial demonstration of CHIP mutations in local tissues of patients with PAD suggested that CHIP may play a role in PAD pathophysiology and that anti-inflammatory therapies targeting CHIP may be useful in treating PAD [84]. It is unclear at what stage of disease these mutations occur, and whether the vascular tissue cells promote inflammation in an endocrine manner, like the HSCs, cause local paracrine inflammation, or both. Further understanding the downstream effects of DNMT-mediated DNA methylation and the role of non-HSC mutations will help shed light on how to target these pathways in atherosclerosis and other vascular diseases. 

CHIP mutations have also been studied in myocardial infarction (MI). Wang et al. found that having *DNMT3A* or *TET2* mutations at a VAF of ≥2% was associated with a significantly increased risk of death and major adverse cardiac events in patients who presented with a ST segment elevation MI [85]. The concentrations of IL-6 and IL-1β were increased in the blood of these individuals, and this inflammatory phenotype was suggested to contribute to a poor prognosis [85]. 

In normal adult cardiac progenitor cells, DNMT3A is responsible for repressing the transcription of Wnt inhibitory factor 1 (Wif1) [86]. Wif1 promotes cardiac progenitor cell differentiation by suppressing *Wnt*, a gene that encodes many secreted proteins which regulate embryonic development and homeostatic regeneration of adult progenitor cells, and which reduce cardiac cell differentiation [86]. DNMT3A-mediated suppression of Wif1 upregulates Wnt activity, reducing differentiation capacity in the adult heart and preventing cardiac remodelling after infarction [86]. Improved heart function post-MI was observed in mice after the injection of cardiac progenitor cells transfected with si-DNMT3A, as DNMT3A downregulation resulted in increased Wif1 expression and subsequent Wnt suppression, thereby promoting cardiac cell differentiation [86]. Further, miR-29a, which is upregulated early in the process of healthy cardiac progenitor cell differentiation, was determined to target and downregulate DNMT3A expression (Figure 5), suggesting that overexpression of miR-29a is a potential therapeutic modality to regulate the DNMT3A-Wnt axis and promote cardiomyocyte regeneration after MI [86]. 

Wang et al. showed that DNMT1-dependent methylation of runt-related transcription factor (RUNX) 3 promotes cardiac microvascular endothelial cell damage and inflammation after acute MI [87]. LncRNA potassium voltage-gated channel subfamily q member 1 overlapping transcript 1 (KCNQ1OT1) was shown to contribute to MI progression by recruiting DNMT1 to the RUNX3 promoter [87]. Knocking down KCNQ1OT1 prevented cardiac microvascular endothelial cell damage and inflammation in mice [87]. Finally, Gambacciani et al. demonstrated that levels of miR-29a and miR-30c, which downregulate DNMT3A (Figure 5), are decreased in cardiac ischemic tissue, while levels of DNMT3A are increased [88]. Reduced levels of these miRs in infarcted tissue could explain the aberrant DNA methylation patterns seen in cardiomyopathies [88]. 

## 7. DNMTs in Other Vascular Pathologies

DNMTs’ dysregulation occurs in a variety of other diseases, including thrombosis. *DNMT3A* mutations driving CHIP have been reported in idiopathic splanchnic vein thrombosis [89], and are associated with a risk of unprovoked pulmonary embolism [90]. Further, the presence of ≥1 CHIP driver mutation (in *DNMT3A*, *TET2*, or *ASXL1*) significantly increased the risk of thrombosis in patients with polycythemia vera [91]. *DNMT3A*-driven CHIP is also associated with stroke [92,93]. Vijay et al. generated a database of potential miRNAs that may regulate DNMTs in thrombotic disorders, suggesting potential targets for thrombosis treatment and biomarker development [94]. Recently, Zhang and Qin demonstrated that in patients with lower extremity deep vein thrombosis, lncRNA LINC00659 facilitated the DNMT3A-mediated methylation (Figure 5) of the fibroblast growth factor 1 (FGF1) promotor, which inhibited the proliferation and angiogenesis ability of endothelial progenitor cells [95]. This indicates a novel DNMT3A-mediated pathway that may be activated to promote lower extremity deep vein thrombosis.

Several other studies have found that DNMTs may also play a role in vascular endothelial dysfunction. Zhao et al. demonstrated, in mice, that transient hyperglycemia directly upregulated DNMT1 expression, causing hypermethylation of *angiopoietin-1* (*ang-1*) and subsequently decreased ang-1 expression [96]. Downregulation of this angiopoietin, a regulator of vascular development and angiogenesis, resulted in a sustained activation of nuclear factor (NF)-kappa-B (NF-κB), a transcription factor that promotes inflammation and immune responses, causing endothelial dysfunction [96] in atherosclerosis [97] and PAH [98]. Consistent with this, the proliferation and migration of endothelial cells in diabetic retinal vascular dysfunction is partially due to the downregulation of circular RNA (circRNA) DMNT3B. CircRNAs are competing endogenous RNAs that mop up miRs. In diabetic retinopathy, loss of circRNA DNAMT3B increases miR-20b-5p expression (Figure 5), promoting a hyperproliferative phenotype [99]. 

Downregulation of DNMT1 may also play a role in Henoch–Schoenlein Purpura, an autoimmune vasculitis affecting the skin, joints, kidneys, and intestines, resulting in a purpuric skin rash, abdominal pain, and hematuria. In Henoch–Schoenlein Purpura, mitogen-activated protein kinase and extracellular signal-regulated kinase (MAPK/ERK) activity appears to be downregulated, resulting in less phosphorylated ERK, which is normally responsible for DNMT1 activation, thereby leading to reduced DNMT1 activity and potential epigenetic dysregulation contributing to the disease phenotype [100]. 

Environmental factors may also trigger DNMT methylation. Prenatal exposure to various air pollutants, including particulate matter, nitrogen dioxide, and ozone, increased the DNA methylation of long interspersed nuclear element-1 (LINE-1) [101]. LINE1 is a retrotransposon that is transcriptionally active in embryogenesis and can be reactivated in adulthood in cancers, such as colorectal and lung cancer [102]. The magnitude of the association between air pollution and LINE1 methylation was altered based on single nucleotide polymorphisms in the *DNMT1*, *DNMT3B*, *TET2*, and thymine DNA glycosylase (*TDG*) genes, and it was concluded that variation in any of these methylation regulators may play a role in the subsequent development of cardiovascular complications [101]. In fact, two single nucleotide polymorphisms (SNPs) in DNMT genes (rs16999714 in *DNMT1* and rs6579038 in *DNMT3B*) increased vulnerability to prenatal ozone-induced alterations in cardiovascular function at 11 years of age [101].

DNMT1 is also relevant to systemic hypertension. The rs2228611 SNP in DNMT1 is associated with susceptibility to essential hypertension in males [103]. Hypertension increases endothelial shear stress in the arterial circulation, which further induces DNMT1-dependent endothelial cell DNA hypermethylation and decreases angiogenesis [104]. 

## 8. DNMTs in Heart Failure

Heart failure is defined as insufficient cardiac output to mediate the body’s needs at rest or insufficient reserve to meet the body’s needs during exercise. It is often associated with congestion in the pulmonary or systemic circulation. DNMTs contribute to the mechanisms of heart failure in several ways: Deng et al. demonstrated that DNMT1 expression was increased in the hearts of rats with doxorubicin-induced left-sided heart failure (LHF) [105]. DNMT1 aggravated LHF by methylating the miR-152-3p promoter and inhibiting miR-152-3p expression (Figure 5), which upregulates the transcription factor ETS protooncogene 1 (ETS1) [105]. ETS1 then promotes the transcription of Ras homolog family member H (RhoH), an atypical GTPase in the RAS family that inhibits mitophagy [105]. The removal of damaged portions of mitochondria (referred to as mitophagy) is critical for maintaining organelle function and cellular homeostasis and inhibiting this process can cause the accumulation of dysfunctional mitochondria, which reduces cardiac function [105,106].

A mechanism by which DNMT1 is upregulated in LHF relates to increased activity of histone deacetylase 3 (HDAC3), which deacetylates DNMT1 to inhibit its degradation via ubiquitination, leading to increased DNMT1 expression [107]. DNMT1 represses the transcription of Src homology domain 2-containing tyrosine phosphatase-1 (SHP-1), a protein-tyrosine phosphatase (PTP) that is anti-apoptotic and cardioprotective [107,108]. Consequently, DNMT1-mediated SHP-1 downregulation promotes cardiomyocyte hypertrophy and apoptosis [107]. DNMT1 expression is also increased in the hearts of mice with either pressure overload-induced LHF or adriamycin-induced LHF [109]. Conversely, the knockdown of DNMT1, using clustered regularly interspaced short palindromic repeats/caspase 9 (CRISPR/Cas9), is cardioprotective [109]. DNMT1 knockout causes protective gene reprogramming by altering gene methylation patterns, further supporting the thesis that upregulated DNMT1 activity contributes to LHF [109]. Another experiment investigating the role of selenium supplementation in an advanced glycation end product (AGE)-induced rat model of LHF showed that both DNMT1 and DNMT2 expression levels were significantly increased in pathological cardiac tissue, and that selenium supplementation could significantly suppress DNMT2 expression [110]. Reduced DNMT2 resulted in decreased DNA methylation of the glutathione peroxidase 1 (GPX1) promoter, enhancing GPX1 expression [110]. GPX1, a regulator of endogenous reactive oxygen species (ROS) generation, could then suppress ROS production and myocyte apoptosis, enhancing the function of cardiac myocytes [110]. However, selenium supplementation did not significantly reduce DNMT1 expression [110]. DNMT3B may play a positive role in LHF prevention. In contrast to the benefits of inhibiting DNMT1 and DNMT2 in LHF, cardiac-specific deletions of DNMT3B resulted in myocardial thinning, fibrosis, and severe systolic insufficiency, causing LHF in a murine model [111]. Additionally, piwi-interacting RNA (piRNA)-6426 overexpression promoted DNMT3B-mediated methylation of the sterol o-acyltransferase 1 promoter, decreasing hypoxia-induced oxidative stress and inflammation in cardiomyocytes, ultimately preventing LHF [112]. These studies demonstrate the importance of adequate DNMT3B function in preventing LHF. 

Monocytes and T cells with *DNMT3A* mutations manifest an inflamed transcriptome that may play a role in LHF [113]. In these cells, *DNMT3A* CHIP mutations upregulate key inflammatory markers, including NLRP3, IL-1β, IL-6, and IL-8 [113]. *DNMT3A* mutations are common in patients with reduced LHF and reduced left ventricular ejection fraction, and are associated with accelerated progression of LHF, both in ischemic and non-ischemic patients [114]. Recently, Assmus et al. found that a novel VAF threshold of only 1.15% (the conventional threshold for the definition of CHIP pathology being 2%) for the *DNMT3A*-CHIP driver mutation is associated with worse outcomes in patients with chronic ischemic LHF [115]. Further identifying the DNMT-mediated inflammatory players involved in heart failure will help reveal novel targets for therapeutic development.

## 9. DNMTs in Cardiac Fibrosis

Upregulated DNMT1 activity can promote cardiac fibrosis. As previously mentioned, in a MCT-induced PAH model, RV fibroblasts experience a HIF-1α-PDK-mediated shift in Warburg metabolism due to increased DNMT1 and decreased miR148b-3p expression, resulting in fibroblast proliferation and increased collagen production in the RV [43]. Xu et al. found that DNMT1-mediated hypermethylation of the miR-152-3p promotor reduces miR-152-3p expression (Figure 5) which upregulates the Wnt1/β-catenin signalling pathway, causing the proliferation of TGF-β-stimulated cardiac fibroblasts from Sprague Dawley rats [116]. Accordingly, reduced DNMT1 methylation upregulates miR-152-3p and inhibits cardiac fibroblast proliferation [116]. miR-369-5p also binds and downregulates DNMT1 activity (Figure 5), preventing suppression of Patched1 and fibroblast activation [117]. Consistent with this, downregulation of miR-369-5p increases DNMT1-mediated hypermethylation of the Patched1 gene, silencing the activity of the Patched1 receptor, a transmembrane protein involved in the hedgehog signalling pathway, ultimately causing fibroblast proliferation and cardiac fibrosis [117]. 

Not only do many miRs regulate DNMT1, DNMT1 mediates the methylation of lncRNA GAS5 (Figure 5), which promotes cardiac fibroblast pyroptosis by increasing caspase 1 and NLRP3 expression [118]. DNMT1 also plays a role in diabetic cardiac fibrosis, as DNMT1-induced hypermethylation of the promoter region of the cytokine signalling 3 (*SOCS3*) gene decreases the expression of SOCS3 [119]. Decreased SOCS3 activates STAT3, activating cardiac fibroblast proliferation [119]. A significant inverse correlation between the expression levels of DNMT1 and SOCS3 has also been seen in the hearts of patients with diabetic cardiac fibrosis [119]. Homocysteine also upregulates DNMT1 in diabetic cardiac fibrosis, leading to the inhibition of androgen receptors and an increase in autophagy of cardiac fibroblasts [120]. Together, this research suggests that DNMT1 promotes cardiac fibrosis, and further illustrates that DNMT1 is both regulated by, and regulates, multiple miRNAs and lncRNAs.

Conversely, He et al. used cardiac fibroblasts harvested from the LVs of Sprague Dawley rats, with infarcted cardiac tissue induced via permanent coronary artery ligation, and found that downregulation of DNMT1-mediated methylation of the α-SMA promoter contributed to cardiac fibroblast differentiation [121]. In contrast to the previously discussed studies, this work suggested that decreased DNMT1 activity might also lead to cardiac fibrosis. However, further investigations are needed to help reproduce and validate this finding [121].

DNMT3A can also promote cardiac fibroblast autophagy and cardiac fibrosis by decreasing the expression of miR-200b (Figure 5) [122]. Supporting this, Qin et al. found miR-200b upregulation decreased DNMT3A expression (Figure 5) and inhibited cardiac fibrosis [123]. Together, this suggested that miR-200b and DNMT3A can regulate the expression of one another. miR-29a was also found to reduce cardiac fibrosis by downregulating the expression of DNMT3A (Figure 5) [124]. Another mechanism by which DNMT3A drives cardiac fibrosis is by downregulating Ras association domain family 1 isoform A (RASSF1A), a tumour suppressor gene that inhibits ERK1/2 activity and aids in protection from cardiac fibrosis [125]. Hypoxia also upregulates hypoxia-inducible factor 1-alpha (HIF-1α) and DNMT3A expression [126]. This mechanism promotes a pro-fibrotic phenotype; conversely, knocking down DNMT3A can prevent hypoxia-induced cardiac fibrosis [126]. Zhao et al. investigated the therapeutic efficacy of low-intensity pulsed ultrasound in attenuating hypoxia-induced cardiac fibrosis [126]. They found that ultrasound energy (0.5 MHz; 100 cycles; 19.30 mW/cm^2^–120.63 mW/cm^2^) reduced cardiac fibrosis in a dose-dependent manner and reversed elevated expression of HIF-1α and DNMT3A [126]. Therefore, like DNMT1, increased DNMT3A activity can also exacerbate cardiac fibrosis. 

Watson et al. employed hypoxia and transforming growth factor β (TGF-β) stimulation to better understand the effects of ischemia on cardiac fibroblast activity. Hypoxia increased the expression of DNMT1, DNMT3B, α-smooth muscle actin (α-SMA), and collagen 1, leading to a pro-fibrotic phenotype [127]. Inhibiting DNMT3B with siRNA decreased the expression of α-SMA and collagen 1, and 5-aza-2′-deoxycytidine attenuated the inflammatory effects of TGF-β, reducing cardiac fibrosis [127]. 

Overall, the upregulation of DNMT1, DNMT3A, and DNMT3B most often promotes cardiac fibrosis, suggesting the therapeutic potential of inhibiting these proteins in preventing the activation and proliferation of cardiac fibroblasts. 

## 10. DNMTs in Cardiomyopathies

DNMTs are also implicated in mediating reduced cardiomyocyte contractility in several cardiomyopathies. *DNMT3A* and *TET2* CHIP mutations have been associated with a statistically insignificant trend towards poor clinical outcomes in patients with non-ischemic dilated cardiomyopathy [128]. DNMT3A is activated in cardiomyocytes experiencing pathological stress in mice caused by transaortic constriction and in humans with LV hypertrophy due to hypertension and/or obesity [129]. DNMT3A plays a role in the hypermethylation and suppression of the motor gene myosin heavy chain 6 (*MYH6*), contributing to impaired cardiac contractility [129]. However, Madsen et al. demonstrated that *DNMT3A*-knockout in cardiomyocytes, derived from human-induced pluripotent stem cells, impaired contraction kinetics, increased lipid accumulation, and compromised glucose metabolism and mitochondrial function [130]. This same group demonstrated that the induction of hypertrophy, using endothelin-1 and phenylephrine, restores glucose metabolism and reduces the metabolic consequences associated with DNMT3A loss in engineered heart tissue [131]. Interestingly, Nührenberg et al. found opposing results in that no significant cardiac pathologies were associated with cardiomyocyte-specific *DNMT3A/DNMT3B* knockout in mice following chronic left ventricular pressure overload induced via transverse aortic constriction [132]. This finding suggests that alternative mechanisms may play an adaptive role in cardiomyocyte protection [132]. It is, however, worth noting that these opposing findings occurred in different models and may not be directly comparable, as reduced metabolic function was seen in cardiomyocytes from human stem cells [130], while no significant pathologies were seen in cardiomyocyte-specific *DNMT3A/DNMT3B* knockout mice [132].

Zhang et al. investigated sepsis-induced myocardial injury and found that the lncRNA small nucleolar RNA host gene 1 (SNHG1) activates DNMT1-mediated methylation (Figure 5) of the promoter region of *B cell lymphoma-2* (*Bcl-2*), an inhibitor of apoptosis [133]. DNMT1-mediated inhibition of Bcl-2 expression promoted inflammation and apoptosis, contributing to myocardial injury in this LPS-induced sepsis model [133]. Fang et al. studied embryonic cardiac myocytes in which DNMT1 had been deleted [134]. They identified approximately 1000 differentially expressed genes, including several cardiac genes that were upregulated (*Myh6*, *Tnnc1*, *Tnni3*, *Tnnt2*, *Nppa*, and *Nppb*) and one that was downregulated (*Cdkn1C*), demonstrating DNMT1′s significance in the regulation of cardiac gene expression and cardiac function [134].

Regarding treatment, Kakoki et al. recently demonstrated that DNMT1, 3A, and 3B mRNA levels were reduced in Akita mice, a model of diabetes mellitus that develops diabetic cardiomyopathy [135]. They found that cyanocobalamin could prevent and reverse cardiomyopathy by adequately restoring DNMT function, leading to reduced transcription of *SOCS1/3* and production of insulin-like growth factor-1 (IGF-1) [135]. Consistent with previously discussed studies regarding the therapeutic potential of DNMT inhibition, Stenzig et al. demonstrated that daily subcutaneous injections of 2 mg of a DNMT inhibitor, *N*-phthalyl-l-tryptophan (RG108), for 4 weeks partially rescued heart function and decreased cardiomyocyte DNA methylation in a transverse aortic constriction-induced rat model of cardiac hypertrophy [136]. Together, these studies demonstrate the role of DNMTs in cardiomyopathies and shed light on potential areas of future research for preventing myopathy-induced heart failure. 

## 11. DNMTs in Other Cardiac Disorders

In patients with recurrent atrial fibrillation, serum levels of DNMT3A and phosphatidylinositol 3-kinase (PI3K) and Akt/Protein Kinase (PI3K-Akt) were positively correlated with the left atrial volumes, while miR-200b was negatively correlated with the left atrial volume [137]. miR-200b levels were also negatively correlated with DNMT3A and PI3K-Akt protein levels, suggesting that DNMT3A may contribute to recurrent atrial fibrillation by downregulating miR-200b and thereby activating PI3K-Akt (Figure 5) [137]. CHIP associated with *DNMT3A* and *TET2* mutations has recently been associated with an enrichment of pro-inflammatory cardiac monocyte-derived macrophages and an increased risk of post-operative atrial fibrillation in patients [138]. Furthermore, *DNMT3A*- and *TET2*-mutant CHIP have been associated with inflammation in severe degenerative aortic valve stenosis and chronic post-ischemic heart failure [139]. 

Santos-Bezerra et al. investigated the risk associated with DNMT1 mutations in diabetes and found that the DNMT1 SNP rs11085721 was associated with a risk of cardiovascular autonomic neuropathy in females with type I diabetes [140]. Lastly, downregulated lncRNA, protein kinase C alpha antisense RNA 1 (PRKCA-AS1) decreases DNMT1-mediated methylation (Figure 5) of the promoter region of protein kinase C alpha (PRKCA), and subsequently increases PRKCA expression in the mitral valves of patients with rheumatic heart disease, suggesting a DNMT-regulated mechanism in valvular heart disease [141]. 

## 12. DNMTs in Congenital Heart Defects and Paediatric CVD

Appropriate DNMT activity is necessary in fetal and childhood development. Several studies have investigated the role of DNMTs and the effects of *DNMT* mutations in congenital heart defects (CHDs) and paediatric CVD. In a study investigating the interplay between genetic variants and maternal factors in the development of obstructive heart defects (OHDs), such as pulmonary valve stenosis and coarctation of the aorta, SNPs in *DNMT3B* were associated with an increased risk of OHD amongst obese women [142]. Li et al. also investigated the association between the development of obstructive heart defects and maternal–fetal genotype interactions [143]. It was found that a haplotype block, an area of the genome containing only a small number of distinct haplotypes, located in the *DNMT3L* gene may have significant maternal–fetal genotype interaction effects that could lead to OHD [143]. 

Joshi et al. found that the expression levels of DNMT3A and DNMT3B were significantly reduced in the blood of patients under the age of 21 with several forms of congenital heart disease, such as ventricular or atrial septal defects and pulmonary stenosis, suggesting that decreased DNMT3 activity and the related genome’s hypomethylation may contribute to the pathogenesis of CHDs [144]. Sheng et al. found that patients with tetralogy of Fallot (TOF) have significantly lower methylation levels of LINE-1 and lower global DNA methylation levels compared to healthy individuals [145]. They found decreased DNMT1, DNMT3A, and DNMT3B mRNA levels in these patients, suggesting that decreased DNMT-mediated methylation could alter the expression of genes responsible for normal heart development [145]. 

Sometimes, these in utero epigenetic alterations may not have implications until later in life. Tanwar et al. exposed pregnant mice to fine particulate matter (particles with diameters of <2.5 μm) to measure the developmental effects of in utero exposure to air pollution [146]. They found that the expression of DNMT1, DNMT3A, and DNMT3B was increased in the hearts of adult mice exposed to particulate matter in utero [146]. These adult mice experienced global cardiac dysfunction due to methylation pattern alterations resulting from this DNMT upregulation [146]. This exemplifies the Barker hypothesis, with fetal exposure to air pollutants increasing adult CVD susceptibility. 

Previous studies have also investigated the role of DNMT SNPs in relation to the risk of CHDs. Lyu et al. discovered that a SNP pair, including rs11892646 located in the *DNMT3A* gene and rs56219526 located in the *MTRR* gene, was associated with a risk of developing a conotruncal heart defect [147]. Further, a study in southeastern Iran found that the AG (heterozygous) genotype at rs6999593 in *DNMT1* was strongly correlated with the susceptibility to and severity of congenital heart diseases, notably ventricular septal defects and TOF, suggesting its potential as a biomarker of disease [148]. Conversely, in a Southern Chinese population, the rs16999593 SNP in *DNMT1* was associated with a decreased risk of transposition of the great arteries [149]. Finally, the *DNMT3B* rs2424913 TT genotype was associated with CHDs, specifically atrial septal defects, in individuals with Down’s syndrome, suggesting that this genotype may be a predisposing factor and biomarker for CHDs in these individuals [150]. 

## 13. DNMTs in Other Paediatric Diseases

Decreased DNMT1 and DNMT3A mRNA levels in peripheral blood mononuclear cells have been associated with DNA hypomethylation and coronary artery aneurysm in Kawasaki disease [151]. In contrast, Wu et al. revealed that DNMT3A is upregulated in infantile hemangioma epithelial cells from patients, while its regulatory miR, miR-206, is downregulated [152]. They also showed that overexpression of miR-206 in xenografted infant hemangioma endothelial cells inhibited DNMT3A activity and suppressed the development of infantile hemangioma in mice, suggesting the potential of miR-206 as a therapeutic agent (Figure 5) [152].

Sasaki et al. established a *Vasa*-Cre-driven transgenic mouse model that constitutively expressed DNMT3A and DNMT3L ectopically after fertilization [153]. Both of these enzymes are important for germline-specific DNA methylation [153]. They found that all mice were born without complications, but died within 20 weeks due to cardiac failure [153]. In total, 549 genes were downregulated in their heart cells due to perinatal hypermethylation [153]. It is evident that DNMTs are important in proper cardiovascular development, and future research should further investigate their role in CHDs and paediatric CVD.

Finally, it is important to note that mutations in *DNMT3A* have been found to cause an overgrowth and intellectual disability syndrome referred to as Tatton–Brown–Rahman syndrome (TBRS) or *DNMT3A* overgrowth syndrome [154]. Common characteristics of TBRS include abnormal height and/or head circumference, obesity, hypotonia, joint hypermobility, and mild-to-severe intellectual disability [155]. From a cardiovascular perspective, atrial and ventricular septal defects, mitral valve prolapse, and aortic dissection are reported features in TBRS [155]. *DNMT3A* mutations in TBRS patients have been found to affect the protein’s functional domains, and it has been suggested this may impair histone binding and domain–domain interactions [154]. Interestingly, of the mutations found in the original report involving 13 individuals, only 2 were also identified in the 167 confirmed somatic *DNMT3A* mutations reported in hematological malignancies in the Catalogue of somatic mutations in cancer (COSMIC) database [154]. Further, Tatton-Brown et al. reported that no patients in their study possessed a *DNMT3A* mutation affecting the Arg882 residue, the target of over 50% of somatic *DNMT3A* mutations found in AML, suggesting that de novo mutations found in overgrowth syndromes differ from the common somatic *DNMT3A* mutations [154]. Conversely, as of the year 2021, approximately 250 TBRS cases had been identified [156], and DNMT3A R882 has emerged as the most common mutation site in TBRS patients [157,158]. In 2017, Hollink et al. reported the first case of AML development in a TBRS patient who was a 15-year-old with a mutation causing an R882C substitution [159]. To date, several additional *DNMT3A* mutations have also been associated with the development of hematological malignancies in both humans [157,158] and mice [157] with TBRS, suggesting germline *DNMT3A* mutations may also promote clonal hematopoiesis and AML. 

## 14. Conclusions

This review discussed the pathogenesis of DNMT dysregulation and CHIP in PAH and several other CVDs. In summary, increased expression of DNMTs in adults is generally pathologic, but genetic mutations or complete knockout of *DNMT* genes are also associated with disease. Further, germline *DNMT* mutations often lead to cellular and tissue dysfunction, while somatic *DNMT* mutations most frequently manifest in HSCs and promote inflammation. CHIP driven by somatic mutations in *DNMT3A* or *TET2* promotes an inflammatory phenotype that is associated with the development of several CVDs. *TET2* mutations contribute to an inflammatory form of idiopathic PAH. We will continue to investigate the role of *DNMT3A* mutations in the development of PAH, particularly associated PAH. There are several mechanisms by which DNMTs are regulated that could be investigated for therapeutic target. The information presented here should be used to help guide subsequent studies in the identification of biomarkers and the development of potential treatments for PAH and CVDs.

## Abbreviations

α-SMA	α-smooth muscle actin
AML	Acute myeloid leukemia
ApoE	Apolipoprotein E
ASL1	Additional sex combs like-1
BER	Base excision repair
CHD	Congenital heart defect
circRNA	Circular RNA
CVD	Cardiovascular disease
CHIP	Clonal hematopoiesis of indeterminate potential
CAD	Coronary artery disease
CpG	Cytosine—phosphate—guanine
DNMTs	DNA methyltransferases
DNMT1	DNA methyltransferase 1
DNMT2	DNA methyltransferase 2
DNMT3A	DNA methyltransferase 3 alpha
DNMT3B	DNA methyltransferase 3 beta
DNMT3L	DNA methyltransferase 3 ligand
ERK	Extracellular signal-regulated kinase
HDAC	Histone deacetylase
HIF-1α	Hypoxia-inducible factor-1 alpha
HSCs	Hematopoietic stem cells
IL-1ß	Interleukin-1ß
lncRNA	Long non-coding RNA
LINE-1	Long interspersed nuclear elements-1
MAPK	Mitogen-activated protein kinase
MCT	Monocrotaline
MDS	Myelodysplastic syndrome
MI	Myocardial infarction
miR	MicroRNA
mRNA	Messenger RNA
mtDNA	Mitochondrial DNA
NF-κB	Nuclear factor kappa-light-chain-enhancer of activated B cells
OHD	Obstructive heart defect
PAD	Peripheral artery disease
PH	Pulmonary hypertension
PAH	Pulmonary arterial hypertension
PASMCs	Pulmonary artery smooth muscle cells
PCR	Polymerase chain reaction
PDK1	Pyruvate dehydrogenase (PDH) kinase 1
PDK3	Pyruvate dehydrogenase (PDH) kinase 3
RRBS	Reduced representation bisulfite sequencing
RVH	Right ventricular hypertrophy
siRNA	Silencing RNA
SNP	Single nucleotide polymorphism
SOD2	Superoxide dismutase 2
TBRS	Tatton–Brown–Rahman syndrome
TET2	Tet methylcytosine dioxygenase 2
TDG	Thymine DNA glycosylase
VAF	Variant allele frequency
VSMCs	Vascular smooth muscle cells
WGBS	Whole-genome bisulfite sequencing
